# Immunotactoid glomerulopathy and chronic lymphocytic leukemia: The need for a multidisciplinary approach

**DOI:** 10.1002/jha2.592

**Published:** 2022-12-13

**Authors:** Angela Rago, Laura Pettorini, Alessandro Andriani, Tommaso Caravita di Toritto

**Affiliations:** ^1^ Haematology Unit, ASL ROMA 1 Santo Spirito Hospital of Rome Rome Italy; ^2^ Nefrology Unit, ASL ROMA 1 Santo Spirito of Rome Rome Italy; ^3^ Internal Medicine Villa Betania Hospital Rome Italy

**Keywords:** chronic lymphocytic leukemia, immunotactoid glomerulopathy

## Abstract

Chronic lymphocytic leukemia (CLL) is the most common type of leukemia in western countries. The association between CLL and glomerular disease (GD) is rare. The most frequent GD associated with CLL is membranoproliferative membranous glomerulonephritis (GN) (MPGN) (45%) types I and II, followed by membranous glomerulonephritis, with the same reports of immunotactoid glomerulopathy (ITG). We report a case of ITG diagnosed on kidney biopsy in a CLL patient and the response of renal parameters to drug treatment for CLL. The patient was treated with several lines of therapies with a good response.

## 1 INTRODUCTION

Chronic lymphocytic leukemia (CLL) is the most common leukemia in the Western world with an incidence of 4.2/100,000/year. The incidence increases to more than 30/100,000/year at an age of >80 years. The median age at diagnosis is 72 years. About 10% of CLL patients are reported to be younger than 55 years [1].

The association between CLL and glomerular disease (GD) is rare. The most frequent GD associated with CLL is membranoproliferative membranous glomerulonephritis (GN) (MPGN) (45%) types I and II, followed by membranous glomerulonephritis (MGN)(20%), with the same reports of immunotactoid glomerulopathy (ITG) [2]. This latter is a term introduced by Schwartz and Lewis to describe a GD characterized by the presence of Congo‐red‐negative organized glomerular deposits that stain for immunoglobulin G (IgG) and complement by immunofluorescence (IF). Often the diagnosis of ITG is limited to patients with hematologic disorders, cryoglobulinemia, or systemic lupus erythematosus (LES) [3]. When cryoglobulinemia and LES are excluded, ITG is a very rare GD encountered in 0.06% of native kidney biopsies. Patients with ITG typically present with proteinuria, hypertension, and hematuria. ITG is associated with lymphoproliferative disorders such as CLL and monoclonal gammopathy. The disease may histologically exhibit diffuse proliferative patterns. In most cases, the deposits contain IgG and C3 and light chain restriction by IF. Ultrastructurally, the glomerular microtubules measure 9–45 mm. The pathogenesis of ITG is currently unknown, but the occurrence of a complete response of GD associated with CLL observed after therapy with alkylating agents, steroids and monoclonal antibodies suggest an immune‐mediated mechanism [4].

**FIGURE 1:**
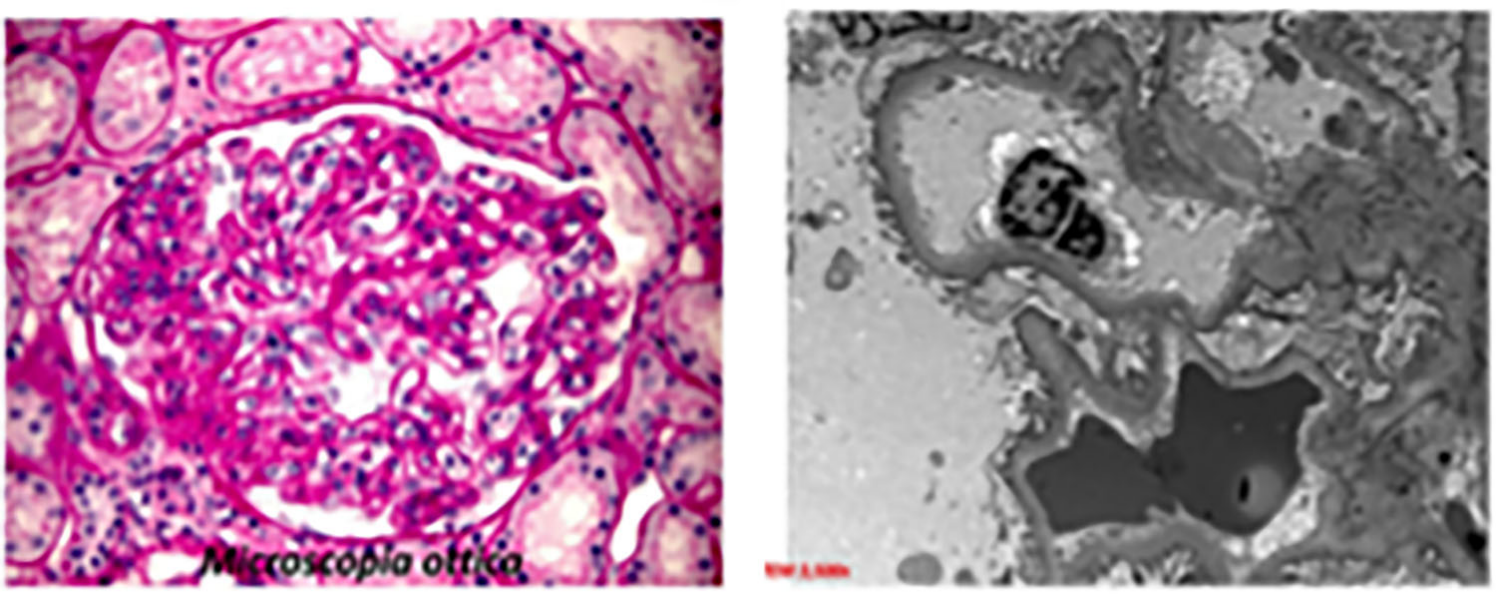
Electron microscopy findings that show a modification of the glomerular loop

**FIGURE 2:**
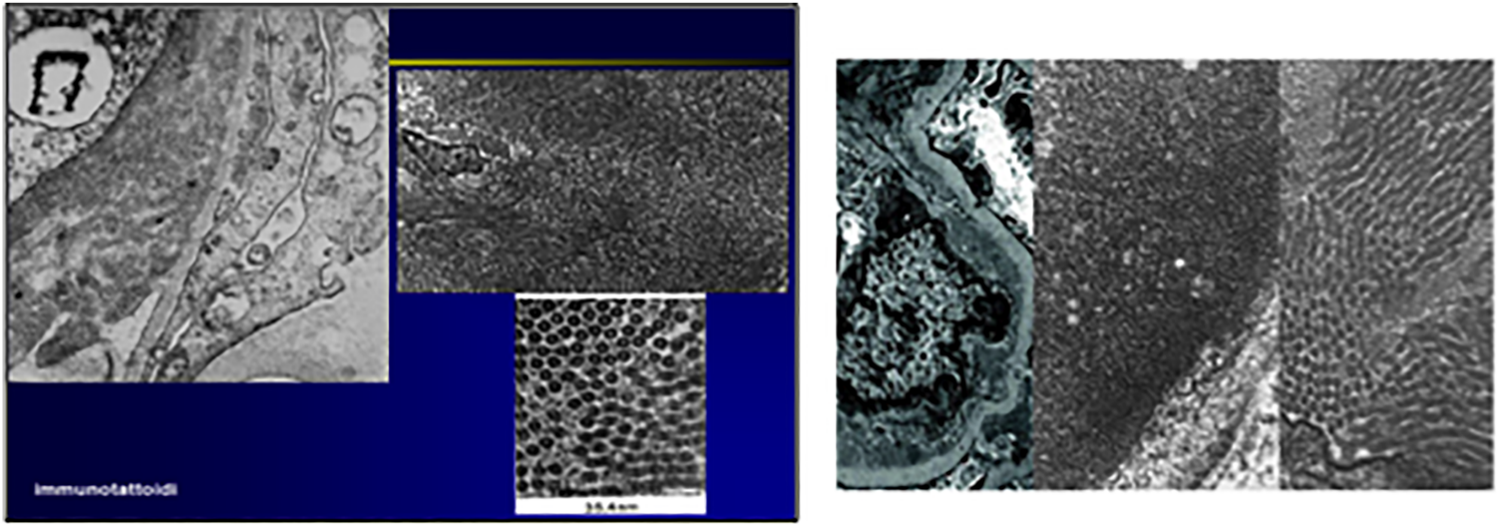
Electron microscopy shows diffuse membranous patterns

We report a rare case of ITG diagnosed on kidney biopsy in a CLL patient and the response of renal parameters to drug treatment for CLL. A 62‐year‐old female presented in 2005 with CLL. In 2012, during follow‐up, the patient presented a urinary total protein (UTP) of 6.9 gr/dl without symptoms. At this time, white blood count (WBC) was 28 × 10^9^/L (lymphocyte count was 12.9 × 10^9^/L). The total body scan (computed tomography [CT]) revealed splenomegaly and abdominal lympho‐adenopathy. The patient underwent to kidney biopsy that showed focal areas of fibrosis and patchy chronic inflammation. IF demonstrated diffusely granular staining for IgG, IgM, C3, C1Q, and lambda light chain, IgA and k light chain were negative. Electron microscopy showed numerous sub‐endothelial deposits with a fibrillary substructure (Figure 1). The fibrils were microtubules with a diameter of 25 mm (Figure 2). Congo‐red was negative. These renal biopsy findings are consistent with ITG. The patient started therapy with chlorambucil 0.2 mg/kg and rituximab every 4 weeks for 6 months and obtained a remarkable response to therapy. At the end of therapy, urinary protein excretion dropped to 210 mg/24 and WBC count was normal (lymphocyte count 1.18 × 10^9^/L). After six years, the patient presented a nephrotic syndrome (NS) progression, in fact, edema and a presence of UTP of 4.5 gr/dl were evidenced. At this time, she was treated with bendamustine and rituximab for 3 cycles interrupted for cardiac complications. After this therapy, the patient performed a follow‐up in 2022, when developed a new increase of UTP (5.4 gr/dl) with edema and normal blood creatinine 0.68 mg/dl. WBC count at this time was 45.4 × 10^9^/L (and lymphocyte count 34.8 × 10^9^/L). The CT scan at this time showed lymphadenopathy with the largest lymph nodes in the abdominal region measuring up to 3 cm. Abdominal effusion was present. She had no type‐B symptoms. On the basis of performance status and previous therapy, the patient started the third line of therapy with chlorambucil and rituximab. After 3 cycles, the UTP was 1.4 gr/dl, WBC was normal 5.5 × 10^9^/L (lymphocyte count 2.4 × 10^9^/L). In addition, the peripheral edema was absent and a CT scan revealed a reduction of abdominal effusion. During the therapy, the patient presented fever and was made a diagnosis with coronavirus disease 2019 (COVID‐19) defined by a positive result on a real‐time polymerase chain reaction assay of a specimen collected on a nasopharyngeal swab [5]. Therefore, the patient stopped chlorambucil and rituximab therapy and started monoclonal antibodies for COVID‐19 infections. The association between GD and CLL is rare despite the relative frequency of this type of leukemia. ITG is a distinctive GD that affects older patients, without no sex predilection. The most common malignancy associated with ITG is CLL. For this reason, the differential diagnosis of proteinuria in CLL patients must be considered ITG. Kidney biopsy is strongly recommended in this patient's setting for differential diagnosis between ITG and other MPGN.

Nasr et al. [6] reported the largest clinic‐pathologic series of 16 ITG patients. Only 3/16 patients had CLL and were treated with steroids, rituximab, and CYT. ITG remission occurred in all three patients after CLL treatment.

In another study, Bridoux et al. [7] described a case series of 23 patients with ITG or MPGN and lymphoproliferative disease (7/23 patients). In all seven cases, the treatment was CYT or corticosteroids alone. Complete or partial reduction of proteinuria was achieved and in 5/7 patients with the lymphoproliferative disease, blood lymphocyte count decreased in the number.

In addition, Abdel‐Raheem [8] describes a case report of a patient with CLL and severe NS and a successful therapy with fludarabine. The author concluded that in this case the use of fludarabine may be considered an efficacious therapy in patients with severe NS and CLL. The author supposed that fludarabine may be considered a reasonable and efficacious alternative to traditional alkylator‐based therapy in patients with severe membranous glomerulopathy with CLL.

In line with all these cases reported in the literature, also in our case, the patient reported a decrease in symptoms and a reduction of proteinuria after the therapy for CLL. In some cases, the treatment of CLL and MPGN with prednisolone, cyclophosphamide, or other therapy produces a good clinical and pathological response. However, it is not possible to distinguish between a direct effect of drug treatment on the mechanism responsible for the GN and the reduction of CLL tumor bulk.

In addition, all the cases reported in the literature, confirm the need for multidisciplinary collaboration between nephrologists and hematologists to better define the nature of proteinuria in these patients.

In conclusion, the ITG that occurred in our patient was successfully controlled and resolved both by specific treatment for CLL. This case report highlights that: i) it is very important to define at baseline the presence of proteinuria in CLL; ii) if proteinuria is present, it is important to define the type of kidney disease, also, when possible, with kidney biopsy; iii) future clinical evaluation is required to define the role of treatment in CLL with ITG.

## CONFLICT OF INTEREST

The authors declare that they have no conflict of interest.

## FUNDING INFORMATION

The authors received no specific funding for this work.

## Data Availability

The data present in this manuscript are present in a database of our data set.
